# CO_2_ activation on pristine and defected honeycomb lattice 2D Fe_2_O_3_ monolayer: A DFT study

**DOI:** 10.1016/j.isci.2026.115890

**Published:** 2026-04-27

**Authors:** Abhishek Dhasmana, Kamal Kumar, Sravendra Rana, Abhishek K. Mishra

**Affiliations:** 1 Applied Science Cluster, School of Advanced Engineering, UPES, Dehradun, Uttarakhand 248007, India

**Keywords:** chemistry, applied sciences, nanomaterials

## Abstract

Two-dimensional (2D) transition metal oxides (TMOs) have emerged as promising catalysts for carbon capture and utilization (CCU) by efficiently converting carbon dioxide (CO_2_) into C^1^ and C^2+^ chemicals and fuels. Here, we have employed density functional theory (DFT) calculations to investigate CO_2_ activation on pristine and defect-engineered 2D Fe_2_O_3_ monolayers (MLs). CO_2_ binds to pristine 2D Fe_2_O_3_ ML with an adsorption energy (E_ads_) of −1.09 eV, indicating moderate chemisorption. The presence of vacancies significantly modifies CO_2_ adsorption behavior. Oxygen (V_O_) and iron (V_Fe_) monovacancies, and O and Fe (V_O-Fe_) divacancy, strengthen CO_2_ binding. Among these, V_O_ exhibit the most favorable activation characteristics. Vibrational frequency analysis reveals a pronounced shift in vibrational modes at the V_O_ site, along with substantial molecular bending (∠O-C-O∼128°), indicating bond weakening and enhanced reactivity. In contrast, V_Fe_ maximizes adsorption strength but stabilizes a nearly linear CO_2_ geometry, limiting activation.

## Highlights

• DFT study of CO_2_ activation on pristine and defected 2D Fe_2_O_3_ monolayers
• Pristine and O-vacancy Fe_2_O_3_ show strong CO_2_ adsorption with bending
• PDOS and Bader analysis show charge transfer from Fe_2_O_3_ to CO_2_
• Fe and Fe-O vacancies show weak interaction; CO_2_ stays linear

## Graphical abstract

**Figure:**
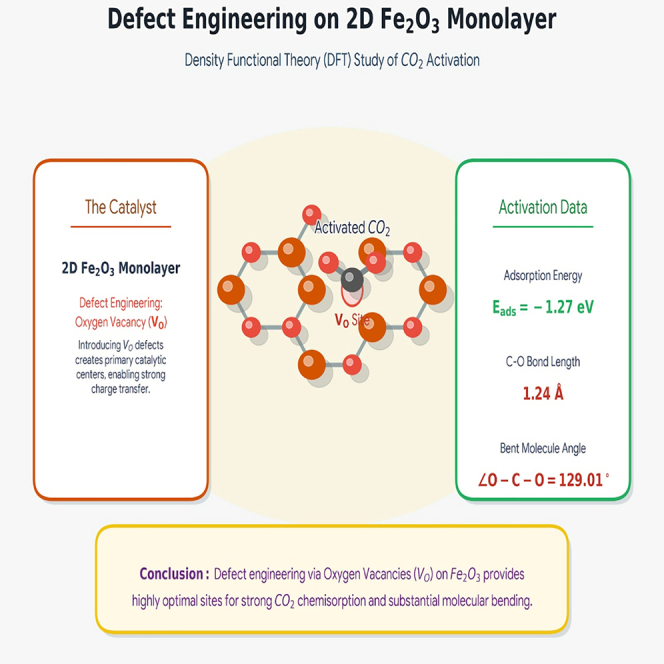

## Introduction

Carbon dioxide (CO_2_) is the primary greenhouse gas driving global warming, with its atmospheric concentration currently 420 ppm (approx.) and still rising due to anthropogenic activities such as fossil fuel combustion and deforestation.^1^ Effective CO_2_ conversion into value-added chemicals or fuels offers a dual benefit, i.e., mitigating greenhouse gas emissions while creating sustainable carbon feedstocks for industrial use.^2^ Technologies for CO_2_ capture and its utilization, such as catalytic conversion, can reduce dependence on non-renewable resources and support circular carbon economies.^3^ Addressing CO_2_ conversion is crucial to meeting international climate targets, as outlined in the Paris Agreement, which aims to limit the global temperature rise.^4^

Transition metal elements, such as gold (Au), silver (Ag), copper (Cu), and so on, have a good ability to activate the CO_2_ molecule and convert it into CO, HCOOH, CH_3_OH, or CH^4^.^5^^,^^6^^,^^7^ Understanding the vibrational properties of CO_2_ upon adsorption is central to evaluating its activation and conversion on catalytic surfaces.^8^ Phonon analysis, whether obtained experimentally through infrared/Raman spectroscopy or theoretically via density functional perturbation theory, provides a direct fingerprint of bond weakening and molecular distortion. The red-shift of the characteristic asymmetric and symmetric stretching modes, along with the altered bending frequency, signals electron transfer into the antibonding 2π∗ orbital of CO_2_, a critical first step toward chemical reduction.^9^ Vibrational softening correlates with geometric bending of the O-C-O moiety and has been widely recognized as the hallmark of activated CO_2_ capable of transforming CO, formate, or other reduced products.^10^ Consequently, phonon analysis not only validates the extent of CO_2_ activation but also bridges the gap between electronic structure, surface chemistry, and catalytic reactivity, offering mechanistic insights into pathways of sustainable CO_2_ conversion. Chernyshova et al. reported that alkali-promoted Cu surfaces, particularly Cu (111) with Na^+^ cations, achieve CO_2_ bending angles as low as 119^°^, forming stable formate anions (HCOO^−^) through electrochemical reduction. This system demonstrates a strong η^2^(C, O) binding mode with significant charge transfer to the CO_2_ molecule.^11^

Two-dimensional (2D) materials^12^ such as Xenes,^13^^,^^14^ MXenes,^15^^,^^16^ and 2D heterostructures^17^ are highly attractive toward gas molecule adsorption and activation due to their large surface area and abundance of exposed active sites. This unique category of materials is promising for not only catalysis^18^ but also for gas sensing,^19^^,^^20^ optoelectronics,^21^^,^^22^ and thermoelectric applications.^23^ Their tuneable electronic structures enable efficient charge transfer to CO_2_, weakening the C-O bonds and promoting activation.^13^^,^^14^^,^^15^ In particular, transition-metal-rich surfaces and oxygen (O) vacancies in MXenes and 2D metal oxides provide strong adsorption and catalytic sites. These features make 2D materials promising candidates for efficient CO_2_ capture and conversion.^24^

2D Transition metal oxides (TMOs) are promising for CO_2_ adsorption and activation due to their partially filled d orbitals, strong metal-oxygen bonds, and tunable electronic characteristics.^25^ Their rich defect chemistry, particularly the ease of forming vacancies and defects, enables effective charge transfer to the CO_2_ molecule and promotes molecular activation, making them suitable model systems for studying CO_2_ adsorption and conversion.^26^ Several studies show that the 2D TMOs exhibit better adsorption and catalytic activity than their bulk form.^25^^,^^26^ Roth et al.^27^ investigated ultrathin Co_3_O_4_ layers of about 1.72 nm thickness, which exhibit CO_2_ reduction 20 times better than its bulk counterpart. According to density functional theory (DFT) investigation, the charge density near the conduction band edge in the ultrathin Co_3_O_4_ layer was significantly enhanced and exhibited greater dispersion compared to bulk Co_3_O_4_, facilitating rapid carrier transport for efficient participation in CO_2_ electrochemical reduction.^27^ Considering the catalytic behavior of ultrathin Co_3_O_4_ and its antiferromagnetic (AFM) properties with a Néel temperature of about 40 K, this work is focused on the catalytic activity of a 2D ML of Fe_2_O_3_.^27^ Pang et al.^28^ theorized the 2D ML of Fe_2_O_3,_ where two adjacent Fe atoms are connected via an O atom and form a honeycomb lattice with 3-fold rotational symmetries.^28^ Further, confirmed its semi-conducting properties and stability with the formation energy of −5.52 eV/atom, a negative sign symbolizes that it is thermodynamically stable and can be synthesized experimentally.^28^ Recently, Vatansever et al.^29^ studied the electronic and magnetic properties of the Fe_2_O_3_ ML in an AFM arrangement at room temperature, and reported the formation energy and cohesive energy of Fe_2_O_3_ measured to be −0.93 and 4.90 eV/atom, respectively, along with the total charge transfer of 1.86 e^−^ to O from Fe.^29^ Etim et al.^30^ reported Fe (110) surfaces induce CO_2_ bending to 121^°^ with exceptionally high electron density transfer (−1.11e^−^ charge) to the molecule.^30^ This extreme activation leads to direct CO_2_ dissociation into CO + O products, with the formation of carbonate species as secondary products.^30^ Introducing defects also helps in the activation of the CO_2_ molecule; recent DFT and experimental studies on ultrathin transition-metal oxide nanosheets confirm that oxygen vacancies dramatically enhance CO_2_ activation and reduction.^30^ Kumar et al.^31^ reported that by creating an O vacancy, the chemisorption increases by 80% as compared to the pristine 2D MgO ML.^31^ Similarly, Sajid et al.^32^ reported that Cu_2_O-TiO_2_ heterostructure with oxygen vacancies exhibits ∼0.09 eV lower barrier for the ∗CO_2_→∗COOH step versus the pristine oxide, indicating the vacancies boost electron transfer into CO_2_ antibonding orbitals.^32^ Li et al.^33^ reported that ultrathin NiO sheets with abundant O vacancies display much higher CO_2_ adsorption and charge-separation efficiency, yielding very high CO evolution rates (∼16.8 μmol/h) with greater than 95% CO selectivity.^33^ Report by Shen et al.^34^ shows investigations of Cu-decorated Ti_0.91_O_2_ single-layer matrices reveal that oxygen vacancies stabilize CO_2_^−^ intermediates and lower the Gibbs free energy for C-H coupling, enabling C_3_H_8_ formation.^34^

In summary, the previous studies on 2D TMOs such as Co_3_O_4_^27^ and NiO^33^ MLs have reported moderate CO_2_ adsorption on their pristine surfaces, mainly governed by surface metal sites, with weak physisorption and limited activation in the absence of defects.^27^^,^^33^ While these MLs demonstrate catalytic potential, strong CO_2_ activation typically requires defect engineering or external modifications.^26^ The Fe_2_O_3_ ML exhibits unique characteristics, including multiple accessible oxidation states of Fe (Fe^2+^/Fe^3+^) and a higher propensity for oxygen vacancy formation.^35^ These features significantly enhance charge transfer and CO_2_ activation at defect sites.^36^ Therefore, Fe_2_O_3_ provides an ideal and representative 2D TMO platform to systematically investigate defect-induced CO_2_ adsorption and activation mechanisms.^36^

In this work, we perform a comprehensive first-principles comparison of CO_2_ adsorption and activation on pristine, Fe-vacancy (V_Fe_), O-vacancy (V_O_), and Fe-O (V_Fe-O_) divacancy sites in a honeycomb lattice 2D Fe_2_O_3_ ML. Beyond adsorption strength, we demonstrate that strong binding does not necessarily translate into effective molecular activation. Notably, while Fe-vacancy and Fe-O divacancy sites exhibit stronger adsorption energies (E_ads_), oxygen vacancies induce more pronounced CO_2_ activation, as evidenced by significant O-C-O bending, C-O bond elongation, and vibrational frequency redshifts. These findings reveal a non-trivial structure-activity relationship and identify oxygen vacancies as optimal activation centers rather than merely strong binding sites.

## Results

### Pristine Fe_2_O_3_ ML

**Figure 1:**
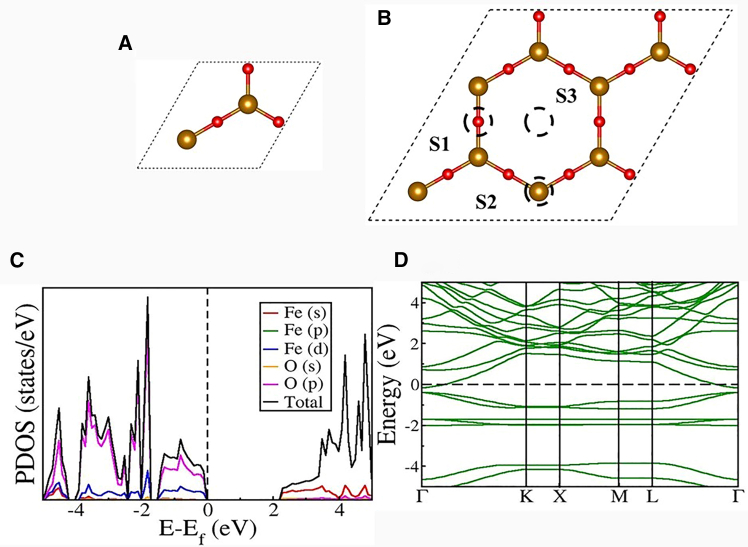
Structural and electronic properties of 2D Fe₂O₃ monolayer (A) Optimized structure of unit cell, (B) 2 × 2×1 super cell, (C) projected density of states (PDOS), and (D) Electronic band structure (EBS) of 2D Fe_2_O_3_. Her, the red color corresponds to the Oxygen atoms, and the brown color corresponds to the Iron atom. S1, S2, and S3 are the sites selected for the interaction study between the CO_2_ molecule and the 2D Fe_2_O_3_ ML.

The geometrical structure of the 2D Fe_2_O_3_ ML adopts a flat and layered configuration, resembling the typical arrangement seen in graphene and other 2D TMOs. The unit cell of this ML contains two iron (Fe) atoms and three oxygen (O) atoms, as shown in Figure 1A. The top view of a 2 × 2×1 supercell is illustrated in Figure 1B, where the periodic lattice of Fe and O atoms is evident. Following full geometric relaxation using DFT, the optimized lattice parameter is found to be a = 6.24 Å, which matches well with the previously reported structure by Pang et al.^28^ The calculated formation energy of −0.93 eV/atom indicated its stability. The Fe-O bond lengths converge to 1.80 Å, and the in-plane O-Fe-O bond angles are stabilized around 120°, confirming the preserved planar geometry with minor corrugation due to Fe’s d-orbital participation in bonding.

Table 1 summarizes the relaxed structural parameters, including bond lengths, bond angles, and lattice constants. Notably, the optimized Fe-Fe interatomic distance is 3.60 Å, which reflects the underlying AFM coupling behavior typical of Fe_2_O_3_ systems. The electronic structure of the optimized geometry was studied using projected density of states (PDOS), as shown in Figure 1C. PDOS reveals that the valence band maximum (VBM) is primarily composed of O 2p orbitals, while the conduction band minimum (CBM) exhibits significant contributions from Fe 3d orbitals, suggesting strong hybridization near the Fermi level (E_f_).

**Table 1:** Calculated values of lattice constants a, b (Å), bond lengths l (Å), and bond angles θ (◦) of the 2D Fe_2_O_3_ML compared with reference earlier DFT studies

Studies	a	b	l	θ
Our work	6.24	6.24	1.80	120
Pang et al.^28^	6.24	6.24	1.80	–
Vatansever et al.^29^	6.18	6.18	–	–

Due to the transition-metal nature of Fe atoms, the partially filled 3d orbitals cause magnetic behavior. The spin-polarized DFT calculations confirm that the ground-state configuration of the Fe_2_O_3_ ML is AFM, with a net zero magnetic moment per unit cell. Each Fe atom has a magnetic moment of about 3.9 μB, while oxygen atoms remain non-magnetic. The calculated band gap was 0.87 eV.

### Defected Fe_2_O_3_ ML

**Figure 2:**
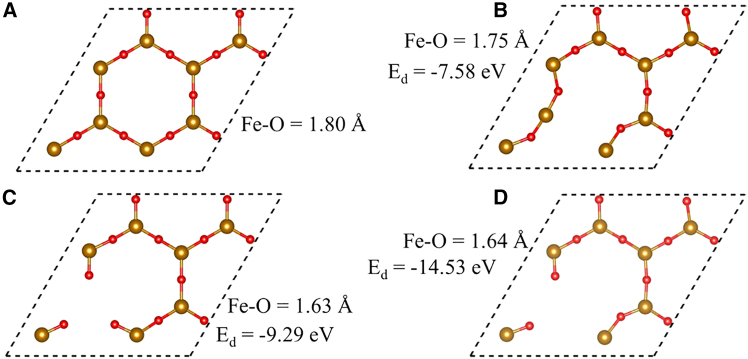
Vacancy defects in 2D Fe₂O₃ monolayer Relaxed structure of a 2×2×1 supercell of (A) pristine, (B) O Vacancy containing, (C) Fe Vacancy containing, (D) O-Fe vacancy containing 2D Fe_2_O_3_ ML. We have given the formation energy of pristine Fe_2_O_3,_ along with defect formation energy (E_d_) values for different defects.

Here, we investigated intrinsic point defects in a 2D Fe_2_O_3_ ML, specifically mono- and di-vacancies, systematically (Figure 2). The defect formation energy, E_d_, was calculated using equation E_d_ = E_P_ − (E_V_ + `E_X_). Here, E_P_, E_V_, and E_X_ denote the total energies of the pristine 2D Fe_2_O_3_ ML, vacancy-defected 2D Fe_2_O_3_ ML, and isolated atom energy of the removed atom, respectively. The calculated E_d,_ provides direct insight into the likelihood of different vacancy types emerging under realistic growth or post-treatment environments. Here, it should be noted that for calculating E_d_, explicit temperature- and pressure-dependent chemical potentials were not considered, as the primary objective of this study is to establish relative defect stability trends within the Fe_2_O_3_ ML rather than to predict absolute defect concentrations under specific synthesis conditions.

In the case of E_d_, the negative sign indicates an exothermic process, implying that defect formation is energetically favorable. A larger magnitude of E_d_ corresponds to a more negative value, signifying higher thermodynamic stability and a greater likelihood of defect formation. Conversely, a smaller magnitude (i.e., less negative or positive) E_d_ indicates a less favorable or endothermic process, suggesting that such defects are less likely to form under equilibrium conditions but may still be realized under non-equilibrium growth conditions or external perturbations. Initially, a single O atom was removed from a 2× 2×1 supercell of the pristine 2D Fe_2_O_3_ ML, followed by full structural relaxation. The computed E_d_ for the O vacancy is found to be -7.58 eV, suggesting this vacancy to be thermodynamically favorable and can persist under ambient conditions. Local lattice distortions surrounding the O vacancy site result in a contraction of adjacent Fe and O bonds from 1.80 Å to 1.75 Å. For comparison, Liu et al. reported the E_d_ for O-vacancy of -2.57 to -3.16 eV for a CeO_2_ ML under 2–5% tensile strain, which is significantly less negative than the value obtained in the present study (-7.58 eV). This comparison indicates that oxygen vacancy formation in the Fe_2_O_3_ monolayer (ML) is thermodynamically more favorable, suggesting a higher propensity for oxygen vacancy stabilization compared to the CeO_2_ ML.^37^

In the case of a Fe vacancy, relaxation again induces a contraction of nearby bond lengths by approximately 9.44%, attributed to atomic rearrangement. The corresponding E_d_ was found to be -9.29 eV, indicating that the formation of this vacancy is energetically more favorable than the O vacancy. The introduction of a divacancy (simultaneous removal of both O and Fe atoms) leads to a further contraction in bond lengths up to 1.64 Å around the cavity. The E_d_ associated with this di-vacancy is calculated to be -14.53 eV, the highest magnitude among all configurations considered, which indicates its highest thermodynamic favorability. Therefore, the relative likelihood of vacancy formation in the ML follows the order: di-vacancy > Fe-vacancy > O-vacancy.

### CO_2_ adsorption on 2D Fe_2_O_3_ ML

To investigate the interaction between the CO_2_ molecule and a pristine 2D Fe_2_O_3_ ML, three distinct adsorption sites were considered. The selection of CO_2_ adsorption sites on the pristine 2D Fe_2_O_3_ ML was guided by the surface symmetry, local atomic coordination, and chemical activity of surface atoms. Accordingly, three high-symmetry adsorption sites were considered: (S1) the top site above a surface Fe atom, representing a metal-centered adsorption environment; (S2) the Fe-O bridge site, capturing cooperative metal-oxygen interactions; and (S3) the hollow site at the center of the hexagonal ring, corresponding to a highly coordinated adsorption region. These sites represent the most distinct and physically relevant adsorption environments on the hexagonal lattice and are widely adopted in previous theoretical studies of CO_2_ adsorption on 2D MLs. The CO_2_ molecule was initially positioned in two different orientations: (1) perpendicular and (2) parallel to the basal plane of the 2D Fe_2_O_3_ ML. Following full structural relaxation, the equilibrium geometries corresponding to these configurations are shown in Figure S1, while the adsorption parameters, including E_ads_, vertical adsorption height (h), molecular bond angle (θ), and relevant bond lengths (L), are summarized in Table S1. E_ads_ was calculated by the equation, E_ads_ = E_sheet+CO2_ - (E_sheet_ + E_CO2_), where E_sheet+CO2_ is the total energy of the CO_2_ adsorbed Fe_2_O_3_ ML, E_sheet_ is the energy of the isolated ML, and E_CO2_ is the energy of an isolated CO_2_ molecule.

**Figure 3:**
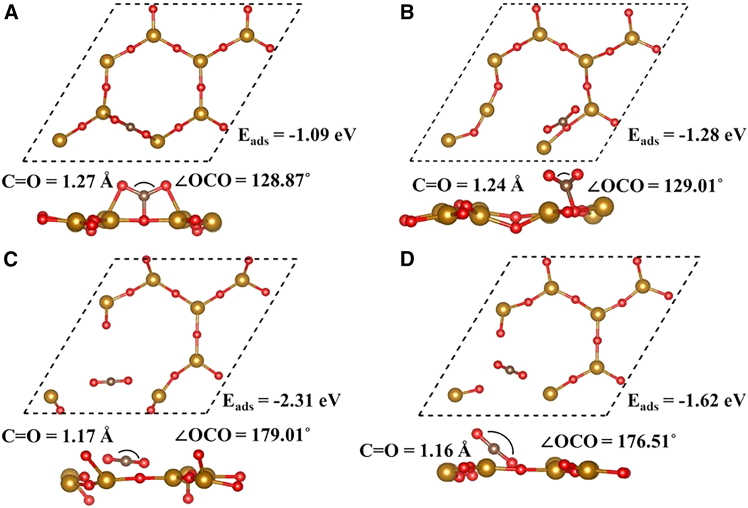
CO₂ adsorption configurations on pristine and defective Fe₂O₃ CO_2_ adsorbed at (A) pristine Fe_2_O_3_, (B) O vacancy, (C) Fe vacancy, and (D) Fe-O vacancy. Maximum adsorption energy is achieved on the Fe-O vacancy, followed by the Fe vacancy, O vacancy, and the pristine Fe_2_O_3_. Despite strong chemisorption on Fe and O-Fe, vacant 2D Fe_2_O_3_ fails to activate the CO_2_ molecule.

The results indicate that both the initial orientation and site selection significantly influence E_ads_. Among all explored configurations, only the most energetically favorable ones are presented in Figure 3. The configuration corresponds to an initial parallel alignment over site 1, resulting in CO_2_ activation with calculated E_ads_ of −1.09 eV at a vertical height of 1.35 Å from the surface. The negative value of E_ads_ confirms the exothermic nature of the adsorption process. Furthermore, the magnitude of E_ads_ exceeds -0.9 eV reported for ZnO-MoSe_2_ ML, implying a stronger interaction with the current ML.^32^ In addition to perpendicular and parallel initial orientations, tilted CO_2_ configurations were also examined at all adsorption sites. Upon full geometry optimization, the initially tilted CO_2_ molecules relaxed back to nearly parallel (horizontal) configurations on the pristine 2D Fe_2_O_3_ ML, indicating that tilted adsorption is not energetically favorable. Here, it should be noted that E_ads_ represents the thermodynamic interaction strength between the 2D Fe_2_O_3_ ML and the CO_2_ molecule at its optimized equilibrium configuration. On the other hand, the activation energy reported in experimental studies corresponds to the kinetic energy barrier that must be overcome for adsorption, surface diffusion, or chemical transformation to occur. While a more negative E_ads_ indicates stronger binding and can qualitatively suggest easier adsorption, it does not directly quantify the activation energy, which requires explicit transition-state calculations. Therefore, the E_ads_ reported here provide insight into the relative stability of CO_2_ adsorption on pristine and defected 2D Fe_2_O_3_ ML, rather than the kinetic rates observed experimentally.

Upon adsorption, the ∠O-C-O bond angle of CO_2_ is reduced from 180° to 128.87°, demonstrating pronounced molecular bending and activation that is characteristic of a chemisorptive interaction rather than weak van der Waals physisorption deactivation.

**Figure 4:**
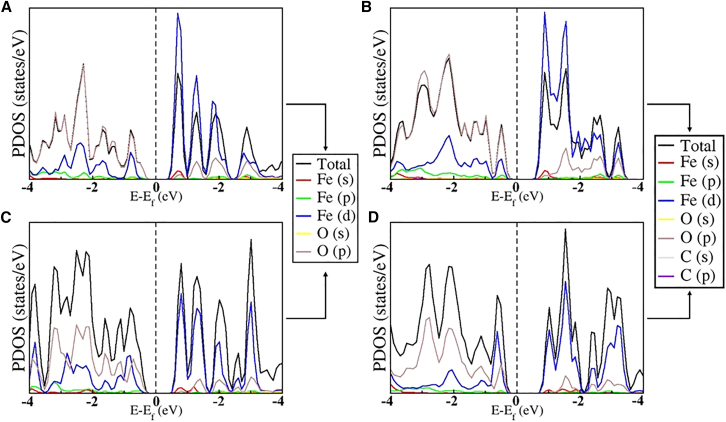
Influence of CO₂ adsorption on the electronic states of 2D Fe₂O₃ Projected density of states (PDOS) plot of the 2D Fe_2_O_3_ ML (2 × 2×1) (A) pristine ML, (B) CO_2_ adsorbed on pristine ML, (C) with oxygen vacancy (V_O_), and (D) CO_2_ adsorbed at V_O_ on 2D Fe_2_O_3_ ML. In Pristine 2D Fe_2_O_3_ and O vacancy 2D Fe_2_O_3,_ the hybridization is seen between Fe 3d and O 2p orbitals, irrespective of the CO_2_ adsorption, which shows that CO_2_ adsorption does not influence the electronic structure of the system. However, some new electronic states can be seen.

We next analyzed the electronic density of state changes as a result of CO_2_ interaction with Fe_2_O_3_ ML. The PDOS of pristine 2D Fe_2_O_3_ ML shows that the valence band region (−4 eV–0 eV) is dominated by O 2p states, while the conduction band (0 eV to +4 eV) mainly consists of Fe 3d states (see Figure 4A). E_f_ lies within the band gap, confirming the semiconducting nature. There is minimal hybridization between Fe and O states near E_f_, which is typical for a pristine oxide. Just after CO_2_ adsorption, new states (see Figure 4B) originating from O p (from CO_2_) appear at the conduction band, indicating weak hybridization with the surface O and Fe atoms.

In the case of the O vacancy, one O atom is absent from the lattice, resulting in the disruption of the hexagonal ring and the formation of unsaturated dangling bonds (see Figure 2). This defect introduces porosity and chemically active sites for adsorption. Multiple geometrical configurations (vertical, horizontal, tilted) of the CO_2_ molecule were explored at the defective site. The three most stable adsorption geometries are illustrated in Figure S2, and their corresponding properties are listed in Table S2.

The most stable configuration, referred to as configuration 2, involves the activation of the CO_2_ molecule above the O atom near the defective site with the E_ads_ of −1.28 eV. Upon relaxation, the molecule undergoes structural deformation, with the bond angle and bond lengths modified to 129.01° and 1.24 Å, respectively. The E_ads_ is significantly more negative than in the pristine case, indicating a transition to strong chemisorption. Introducing an O vacancy significantly modifies the electronic structure. The vacancy generates localized defect states within the band gap, largely derived from undercoordinated Fe 3d orbitals (see Figure 4C). The PDOS shows (see Figure 4D) that the O vacancy provides localized Fe sites with unsaturated bonds at the conduction band. This interaction at the V_O_ site demonstrates that O vacancies are highly favorable for CO_2_ chemisorption and activation, unlike the weak interaction observed on the pristine surface.

Similar behavior is observed in the Fe vacancy on a 2D Fe_2_O_3_ ML, where removal of one Fe atom creates additional dangling bonds. The most stable configuration 3 corresponds to a parallel alignment over the defect site (Fe vacancy), with one terminal atom of the molecule oriented toward reactive lattice atoms of the 2D Fe_2_O_3_ ML. This leads to molecular bending from 180° to 179.01°, and elongation of the CO_2_ molecule bonds by 0.86%. The E_ads_ in this configuration reach −2.31, which is −1.03 eV more negative than that of the O vacancy system, confirming even stronger binding. All the configurations (vertical, horizontal, tilted) of the CO_2_ molecule were explored at the defective site. The three most stable adsorption geometries are illustrated in Figure S3, and their corresponding properties are listed in Table S3.

Finally, the divacancy system O-Fe vacancy was analyzed using a large cavity caused by the vacancy as the interaction region. Upon relaxation, chemical adsorption occurs, with a computed E_ads_ of −1.62 eV, where one terminal atom of the CO_2_ molecule faces a lattice atom, and the second is positioned above the cavity in a tilted orientation. The E_ads_ falls between those of the two previous defect systems, with other parameters such as bond angles and bond lengths. Compared to the Fe vacancy, the molecule is slightly more bent (by 3.5°), and the bond elongation is also reduced (by 0.01 Å), suggesting a relatively moderate activation level.

Overall, among all configurations considered, the 2D Fe_2_O_3_ with Fe vacancy exhibits the strongest interaction with CO_2_, both structurally and electronically. Detailed geometries and properties of the best three relaxed structures of CO_2_ adsorption of di-vacancy are provided in Figure S4 and Table S4, while a comparative summary of the most stable configurations is compiled in Table 2. To directly validate the charge transfer mechanism, we have performed the Bader charge analysis, revealing positive charges of +0.308 e^−^ and +0.258 e^−^ on CO_2_ adsorbed at pristine and oxygen vacancy (Vo) sites, respectively. The positive sign indicates electron depletion from CO_2_, demonstrating that electron transfer occurs from CO_2_ to the Fe_2_O_3_ ML, opposite to conventional CO_2_ activation mechanisms on metallic catalysts. This reverse transfer is attributed to the electron-deficient Fe^+3^ sites in Fe_2_O_3_ acting as electron acceptors rather than donors. The reduced positive charge at Vo sites (+0.258 e^−^) compared to pristine (+0.308 e^−^) indicates that O vacancies provide localized electron density that partially offsets the oxidizing character of neighboring Fe^+3^ sites, correlating with enhanced E_ads_ (−1.27 eV vs. −1.09 eV). Despite reverse electron transfer, CO_2_ activation still occurs through electrostatic polarization and orbital hybridization, evidenced by molecular bending (θ ≈ 129°) and C-O bond elongation. This unique activation pathway reveals Fe_2_O_3_-specific surface chemistry distinct from traditional metal catalysts and provides insights for designing oxide-based CO_2_ conversion catalysts.

**Table 2:** Calculated adsorption energy E_ads_ (eV), adsorption height h (Å), bond lengths l (Å), and bond angle θ, of the most stable CO_2_ configurations at pristine and defected 2D Fe_2_O_3_ MLs

System	E_ads_	h	l		θ
			Fe-O	C-O	
CO_2_ @ Pristine	−1.09	1.35	2.01	1.27	128.87
CO_2_ @ V_O_	−1.27	1.35	2.06	1.24	129.01
CO_2_ @ V_Fe_	−2.31	0.93	1.63	1.17	179.01
CO_2_ @ V_O-Fe_	−1.62	1.06	1.77	1.16	176.51

### Geometric activations of CO_2_ on 2D Fe_2_O_3_ from phonon analysis

**Figure 5:**
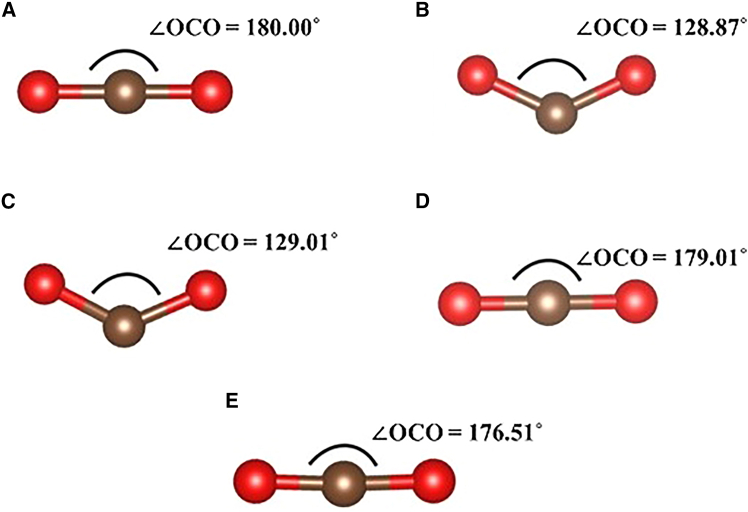
Structural distortion of CO₂ on 2D Fe₂O₃ monolayer Geometry of CO_2_ at (A) gas phase, (B) pristine Fe_2_O_3_, (C) V_O_, (D) V_Fe_, and (E) V_O-Fe_ defected 2D Fe_2_O_3_ ML. Considering the change in bond angle on pristine and O vacancy 2D Fe_2_O_3_, a pronounced angular distortion occurs (∠OCO ≈129°), evidencing substantial molecular activation beneficial for CO_2_ conversion.

In the free state, CO_2_ is a linear, centrosymmetric molecule (O–C–O = 180°) with two equivalent C–O bonds (1.16 Å) (see Figure 5A). Its vibrational spectrum is well characterized by three fundamental modes: the bending mode (ν_b_ ≈ 645 cm^−1^), the symmetric stretch (ν_s_ ≈ 1309 cm^−1^), and the asymmetric stretch (ν_as_ ≈ 2325 cm^−1^). These frequencies reflect strong C=O bonds and the absence of permanent dipole moments due to its D∞h symmetry. Upon adsorption on 2D Fe_2_O_3_, particularly at defect sites, CO_2_ undergoes substantial geometric distortion, the O-C-O bond angle decreases, and C=O bonds elongate asymmetrically. This transformation, often described as CO_2_ activation, which both lowers the bond force constants and lifts the molecular symmetry to C_2_v. Such distortions leave distinct fingerprints in the phonon spectrum, allowing one to quantitatively probe the degree of activation (see Table 3).

**Table 3:** Vibrational modes (bending ν_b_, symmetric stretch ν_s_, and asymmetric stretch ν_as_) of CO_2_ molecule in its gas phase and when adsorbed at pristine, oxygen vacancy (V_O_), iron vacancy (V_Fe_), and di vacancy (V_O-Fe_) sites of 2D-Fe_2_O_3_

System	υ_b_ (cm^−1^)	υ_s_ (cm^−1^)	υ_as_ (cm^−1^)
CO_2_	645	1309	2324
CO_2_ @ Pristine	754	1051	1401
CO_2_ @ V_O_	754	1039	1383
CO_2_ @ V_Fe_	639	1317	2341
CO_2_ @ V_O-Fe_	649	1306	2336

For CO_2_ bound to the pristine Fe_2_O_3_ ML, a dramatic shift of the vibrational spectrum is observed. The asymmetric stretch red-shifts from 2324 cm^−1^ (gas phase) to 1401 cm^−1^, a reduction of nearly 40% (see Table 3). This large decrease directly indicates a significant population of the antibonding 2π∗ orbital, weakening the C=O bonds and bending the O-C-O angle (see Figure 5B). Simultaneously, the symmetric stretch decreases from 1309 cm^−1^ to 1051 cm^−1^, reflecting the reduced restoring force of the equivalent C=O bonds. Interestingly, the bending mode shifts upward from 645 to 753 cm^−1^. This blue shift of ν_b_ is characteristic of bent CO_2_ species, as the molecule departs from linearity, the restoring force for angular deformation increases, thereby stiffening the bending vibration. Together, these shifts confirm that adsorption on pristine Fe_2_O_3_ induces a bent, strongly activated CO_2_ geometry with weakened C-O bonds.

The role of defects becomes clear while investigating adsorption at an O vacancy. At V_O_, CO_2_ exhibits moderate softening of both symmetric and asymmetric stretches, ν_s_ drops to 1039 cm^−1^, and ν_as_ to 1383 cm^−1^. These frequencies represent a noticeable reduction relative to gas-phase CO_2_, suggesting appreciable weakening of the C=O bonds (see Table 3). The vibrational spectrum supports that the molecule deviates from linearity and can approach a carbonate-like configuration (see Figure 5C). In this scenario, the bending mode shifts from 645 cm^−1^ (gas-phase) to 754 cm^−1^, showing a blue shift rather than a softening. This indicates that, at the O vacancy, the bending deformation becomes more energetically demanding, consistent with the molecule being stabilized in a more activated, non-linear geometry. Such a signature often reflects strong charge transfer and the formation of CO_2_^δ−^ species stabilized by defect states of Fe_2_O_3_.

Adsorption at Fe vacancies yields a different picture. Here, ν_as_ remains close to the free CO_2_ value (2341 cm^−1^ vs. 2324 cm^−1^), indicating that the antibonding 2π∗ orbital is not significantly populated. The symmetric stretch (1317 cm^−1^) and bending mode (639 cm^−1^) are likewise only slightly perturbed (see Figure 5D). These data suggest that CO_2_ at V_Fe_ is physiosorbed or only weakly chemisorbed, with negligible electronic activation. The near-preservation of vibrational frequencies implies that the molecule retains its quasi-linear geometry. The combined vacancy site (V_O-Fe_) presents an intermediate case. Both ν_s_ (1306 cm^−1^) and ν_as_ (2336 cm^−1^) remain close to gas-phase values, while ν_b_ increases modestly to 649 cm^−1^. This suggests that although the dual vacancy alters the local coordination environment, the charge transfer into the CO_2_ 2π∗ orbital is minimal, and the molecule largely retains its linear, inactivated geometry (see Figure 5E).

## Discussion

We conducted first-principles-based DFT calculations to investigate the CO_2_ adsorption on both pristine and three defect-engineered, O vacancy, Fe vacancy, and O-Fe vacancy, 2D Fe_2_O_3_ MLs. Adsorption energies indicate that CO_2_ undergoes chemisorption on the pristine surface, with an adsorption energy of −1.09 eV, whereas all three vacancy-containing systems exhibit even stronger chemisorptive binding (E_ads_ = −1.27 eV, −2.31 eV, and −1.62 eV, respectively) accompanied by pronounced molecular deformation of the CO_2_ molecule. Among these, the Fe vacancy demonstrated the most favorable catalytic attributes, owing to its highest adsorption capacity. These findings suggest that strategic defect introduction can markedly enhance the activation and conversion potential of the ML for carbon-capture and utilization (CCU) applications. We anticipate that this study will inform the rational design of 2D catalysts for sustainable energy and chemical production. The structural analysis further reveals that adsorption on the pristine and O vacancy surfaces induces the substantial bending of CO_2_ (∠O-C-O ≈ 128–129°), whereas Fe-related vacancies preserve a nearly linear geometry (∠O-C-O ≈ 176–179°). This bending is directly correlated with phonon softening, where the symmetric and asymmetric stretching modes undergo significant redshifts, most prominently on the V_O_ site (ν_s_ = 560.75 cm^−1^, ν_as_ = 1184.43 cm^−1^), indicating weakening of the C=O bonds and enhanced molecular activation. In contrast, Fe and O-Fe vacancy sites exhibit vibrational features closer to those of gas-phase CO_2_, consistent with weaker activation despite strong adsorption. Taken together, these findings establish a clear structural property correlation: O vacancies are most effective in activating CO_2_ through geometric distortion and vibrational softening, while Fe vacancies maximize adsorption strength but favor the stabilization of the nearly linear molecule. This duality underscores the importance of defect engineering in tuning both binding energetics and activation pathways. We anticipate that these insights will inform the rational design of 2D Fe_2_O_3_-based catalysts for CCU, with broader implications for sustainable energy and chemical production. While the present work focuses on CO_2_ adsorption and activation, these results provide a crucial foundation for exploring subsequent reaction steps. Future studies will extend this framework to investigate reaction intermediates, transition states, and activation barriers for CO_2_ conversion pathways on defect-engineered 2D Fe_2_O_3_ MLs, enabling a comprehensive understanding of catalytic kinetics and reaction mechanisms.

### Limitations of the study

Despite providing useful insights into the CO_2_ activation on pristine and defective 2D Fe_2_O_3_ MLs, the present study has a few limitations. First, the analysis is based on idealized computational models under vacuum conditions, which may differ from realistic catalytic environments, such as temperature, pressure, and solvent effects. Second, only a limited set of point defects (Fe and O monovacancies and the Fe-O divacancy) was considered. In contrast, other possible defects, dopants, or edge sites could influence the activation behavior. Furthermore, the study focuses mainly on adsorption characteristics and electronic structure (PDOS and Bader charge transfer) without exploring the full reaction pathways or energy barriers for subsequent CO_2_ reduction steps. Experimental validation is therefore required to confirm the predicted activity.

## Resource availability

### Lead contact

Further information and requests for resources should be directed to and will be fulfilled by the lead contact, Abhishek Kumar Mishra (akmishra@ddn.upes.ac.in).

### Materials availability

This study did not generate any new materials. All the data were obtained through first-principles calculations.

### Data and code availability

• All data reported in this paper will be shared by the lead contact upon request.
• This study did not generate original code.
• Any additional information required to reanalyze the data reported in this paper is available from the lead contact upon reasonable request.

## Acknowledgments

A.K.M. and A.D. acknowledge the 10.13039/501100001843Anusandhan National Research Foundation (ANRF) for the SURE grant (SUR/2022/004935). AKM also acknowledges 10.13039/100017603UPES for the SEED grant for computational resources.

## Author contributions

Conceptualization, A.K.M.; methodology, A.K.M.; investigation, A.D. and K.K.; writing – original draft, A.D. and K.K.; writing-review and editing, A.K.M., A.D., and K.K.; supervision, A.K.M.

## Declaration of interests

The authors declare no competing interests.

## Declaration of generative AI and AI-assisted technologies in the writing process

During the preparation of this work, the authors used ChatGPT (OpenAI) to check english. The authors reviewed and edited the content and take full responsibility for the publication.

## STAR★Methods

### Key resources table

**Table:** 

REAGENT or RESOURCE	SOURCE	IDENTIFIER
**Software and algorithms**		

DFT Calculations^38^	VASP^39^	https://www.vasp.at/
Exchange-correlation functional (GGA-PBE)^40^^,^^41^	Perdew–Burke–Ernzerhof	Standard functional
Pseudopotentials	VASP PAW^42^ dataset	As supplied with VASP
Visualization Softwares	VESTA/Xcrysden	Standard Tools
DFT+U^28^ Methods	Dudarev approach	Standard implementation in VASP
van der Waals correction (DFT-D3)^43^	Grimme method	Standard implementation
Bader charge analysis	Henkelman group	https://theory.cm.utexas.edu/henkelman/code/bader/

### Method details

A plane-wave energy cutoff of 450 eV was selected for the expansion of wave functions, as determined from convergence tests on total energy and structural stability. For structural relaxation, a Monkhorst-Pack k-point mesh of 4 × 4×1 was applied for the sampling of the Brillouin zone of the primitive unit cell, ensuring convergence of forces and stress within acceptable limits. The convergence criteria for electronic self-consistency were set to 1 × 10^−5^ eV, while the atomic positions were optimized until the Hellmann–Feynman forces on each atom were less than 0.01 eV/Å. To treat the strong on-site Coulomb interaction of Fe 3days electrons, the DFT+U method within the Dudarev approach was employed. A Hubbard U value of 3.0 eV was applied to the Fe 3days states, consistent with previous DFT studies on Fe_2_O_3_ that reported good agreement with structural and electronic properties.^28^ The defect formation energy (E_d_) and the adsorption energy (E_ads_) of a CO_2_ molecule on both pristine and defect-engineered MLs are computed using Equations 1 and 2, respectively.

(Equation 1) $$E_{d}=E_{P}-\left(E_{V}+E_{X}\right)$$

(Equation 2) $$E_{ads}=E_{sheet+{CO}_{2}}-\left(E_{sheet}+E_{{CO}_{2}}\right)$$

Here, E_P_, E_V_, and E_X_ denote the total energies of the relaxed pristine 2D Fe_2_O_3_ ML, vacancy-defected 2D Fe_2_O_3_ ML, and isolated atom energy of the removed atom, respectively. E_sheet+CO2_ represents the total energy of the 2D Fe_2_O_3_ pristine (or defective) ML after CO_2_ adsorption, E_sheet_ is the total energy of 2D Fe_2_O_3_ pristine (or defective ML) before CO_2_ adsorption, while E_CO2_ corresponds to the total energy of the isolated CO_2_ molecule simulated in a supercell.

### Quantification and statistical analysis

VASP^39^ software package has been used to perform DFT-based calculations.
