# Supplementary material for: CO2 activation on pristine and defected honeycomb lattice 2D Fe2O3 monolayer: A DFT study
Source: iScience. 2026 Apr 27;29(6):115890. doi: 10.1016/j.isci.2026.115890 (PMC13226928; doi:10.1016/j.isci.2026.115890)
Supplement: Document S1. Figures S1–S4 and Tables S1–S4 [file mmc1.pdf]

iScience, Volume *29*

Supplemental information

CO_2 activation on pristine and defected honeycomb lattice 2D Fe_2O_3 monolayer: A DFT study

Abhishek Dhasmana, Kamal Kumar, Sravendra Rana, and Abhishek K. Mishra

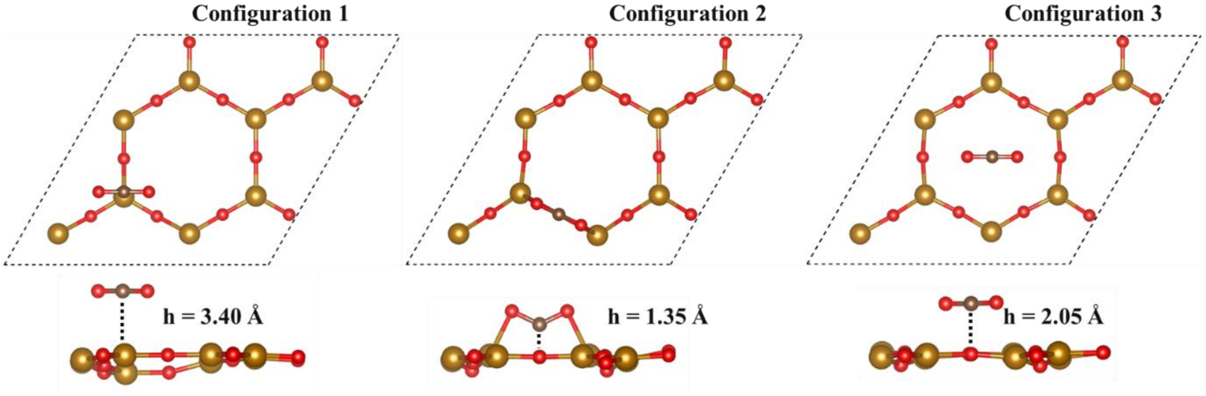

Figure S1. Different configurations of CO_2 adsorption on a pristine Fe_2O_3 monolayer. (see **Table S1** for more details)

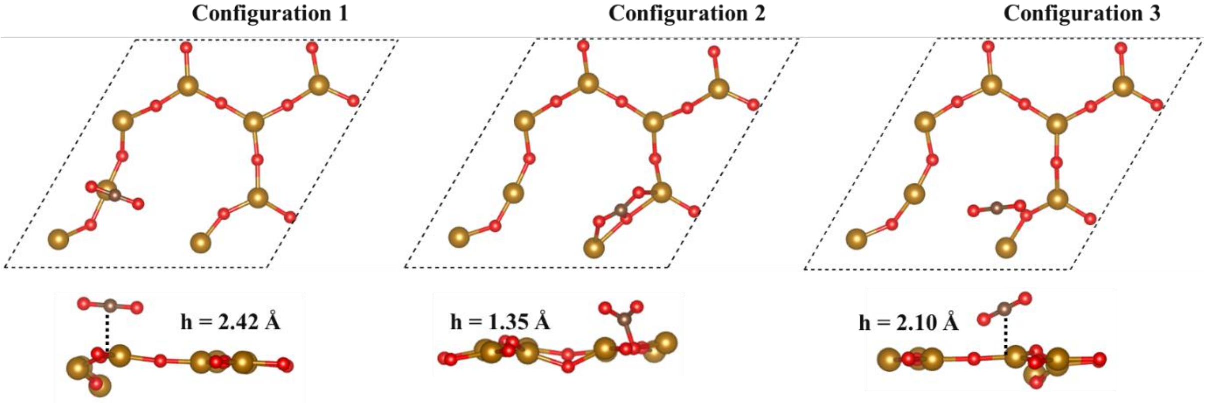

Figure S2. Different configurations of CO_2 adsorption on O vacancy (V_O) Fe_2O_3 monolayer. (see **Table S2** for more details)

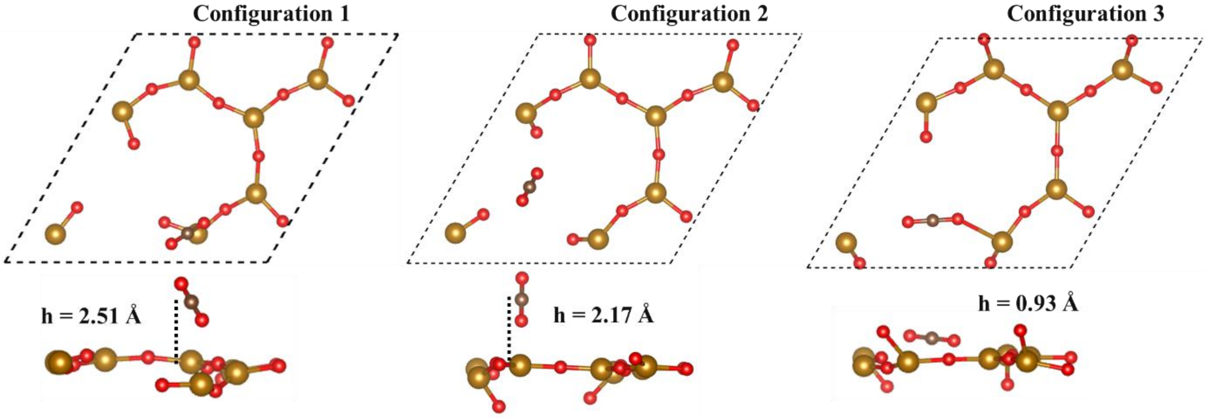

Figure S3. Different configurations of CO_2 adsorption on Fe vacancy (V_{Fe}) Fe_2O_3 monolayer. (see **Table S3** for more details)

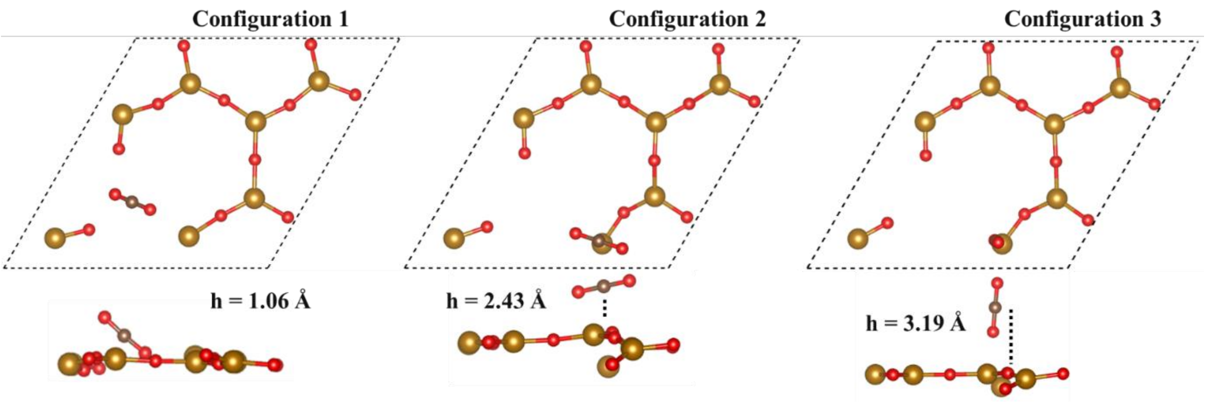

Figure S4. Different configurations of CO_2 adsorption on a di- vacancy (V_{O-Fe}) Fe_2O_3 monolayer. (see **Table S4** for more details)

Table S1. CO_2 adsorption on pristine Fe_2O_3 monolayer.

Configuration	Energy (eV)	Height (Å)	C-O (Å)	∠OCO (º)	Fe-O (Å)
1	-0.09	3.40	1.17	179.48	1.79
2	-1.09	1.35	1.27	128.87	2.01
3	-0.22	2.05	1.17	178.18	1.79

Table S2. CO_2 adsorption on defected (O vacancy) Fe_2O_3 monolayer.

Configuration	Energy (eV)	Height (Å)	C-O (Å)	∠OCO (º)	Fe-O (Å)
1	-0.27	2.42	1.17	179.99	1.79
2	-1.28	1.35	1.27	129.01	2.06
3	-0.29	2.10	1.17	178.89	1.80

Table S3. CO_2 adsorption on defected (Fe vacancy) Fe_2O_3 monolayer.

Configuration	Energy (eV)	Height (Å)	C-O (Å)	∠OCO (º)	Fe-O (Å)
1	-0.39	2.51	1.18	179.11	1.79
2	-0.43	2.17	1.17	179.36	1.79
3	-2.31	0.93	1.17	179.01	1.63

Table S4. CO_2 adsorption on defected (O-Fe vacancy) Fe_2O_3 monolayer.

Configuration	Energy (eV)	Height (Å)	C-O (Å)	∠OCO (º)	Fe-O (Å)
1	-1.62	1.06	1.17	176.51	1.76
2	-0.19	2.43	1.17	179.59	1.77
3	-0.38	3.19	1.17	179.89	1.77
